# Neutrophil-Mediated Phagocytosis of *Staphylococcus aureus*

**DOI:** 10.3389/fimmu.2014.00467

**Published:** 2014-09-26

**Authors:** Kok P. M. van Kessel, Jovanka Bestebroer, Jos A. G. van Strijp

**Affiliations:** ^1^Medical Microbiology, University Medical Center Utrecht, Utrecht, Netherlands

**Keywords:** neutrophil, phagocytosis, *Staphylococcus aureus*

## Abstract

Initial elimination of invading *Staphylococcus aureus* from the body is mediated by professional phagocytes. The neutrophil is the major phagocyte of the innate immunity and plays a key role in the host defense against staphylococcal infections. Opsonization of the bacteria with immunoglobulins and complement factors enables efficient recognition by the neutrophil that subsequently leads to intracellular compartmentalization and killing. Here, we provide a review of the key processes evolved in neutrophil-mediated phagocytosis of *S. aureus* and briefly describe killing. As *S. aureus* is not helpless against the professional phagocytes, we will also highlight its immune evasion arsenal related to phagocytosis.

## Introduction

*Staphylococcus aureus* is a human commensal but also a common course of serious infections, ranging from mild skin infections to more serious life-threating wound and bloodstream infections (1). The innate immunity is an important part of the host defense in elimination of infections caused by *S. aureus*. Notably, many virulence factors of *S. aureus* are directed toward elements of the innate immune defense including its principal phagocyte, the neutrophil (2–4).

In the human blood, neutrophils are the predominant phagocytic cell type, accounting for 50–60% of all leukocytes. The acknowledgment for professional phagocytes started with the first description of motile cells capable of engulfing other matter by Ilya Ilyich Mechnikov, a Russian biologist, best known for his pioneering research on the immune system. Mechnikov received the Nobel Prize in Medicine, jointly with Paul Ehrlich, in 1908 for his work on phagocytosis, which is defined as the uptake of bacteria, parasites, dead host cells, and foreign debris. In addition to neutrophils, dendritic cells, monocytes, and macrophages are considered professional phagocytes, and all cell types are critical in controlling bacterial infection, all be it through different means.

Neutrophils are ready-to-go cells, show a fast response, and have a generally believed short half-life of <7 h. Recent *in vivo* labeling studies, however, estimated the lifespan of neutrophils to be much longer, i.e., 5.4 days (5). Neutrophils are rapidly mobilized from the bone marrow into the circulation, and several subtypes are now characterized based on differential surface antigen expression and function in innate immunity (6–8). The last decade, the role of neutrophils in several other aspects of immunity is appreciated as it has become clear that neutrophils also participate in processes associated with the adaptive immunity and tumor immunology. They display cross talk with adaptive immune cells, i.e., dendritic cells, lymphocytes, and natural killer cells, through secretion of cytokines and reactive oxygen species (ROS), and they interact directly with cells of adaptive immunity via cell surface molecules (9), functions that are most likely associated with different subpopulations or activation states (10, 11). As neutrophils are circulating cells, they first need to leave the bloodstream via diapedesis to reach the site of infection through directed migration along an increasing gradient of chemoattractants, which are derived from bacteria, generated via complement activation or secreted by activated cells including leukocytes (12). For efficient phagocytosis, bacteria need to be covered with opsonins provided by specific immunoglobulins (Igs), the complement system, and others. Uptake of bacteria leads to full activation of the anti-microbial arsenal of the neutrophil leading to killing of the ingested bacteria. The neutrophil is equipped with two major pathways for killing, generation of ROS, and degranulation of granules packed with proteases and specific anti-microbial peptides. The active phagocytosis by neutrophils is eventually followed by a more passive form of elimination of the micro-organisms as the lifetime of the cell is consumed through the formation of neutrophil extracellular traps (NETs) that consist of chromatin and granule content (13). It should be noted that neutrophils and its arsenal of anti-microbials employed to fight infection, sometimes turn against the host itself causing inflammation (14).

## Opsonization and Recognition of *S. aureus*

**Figure 1:**
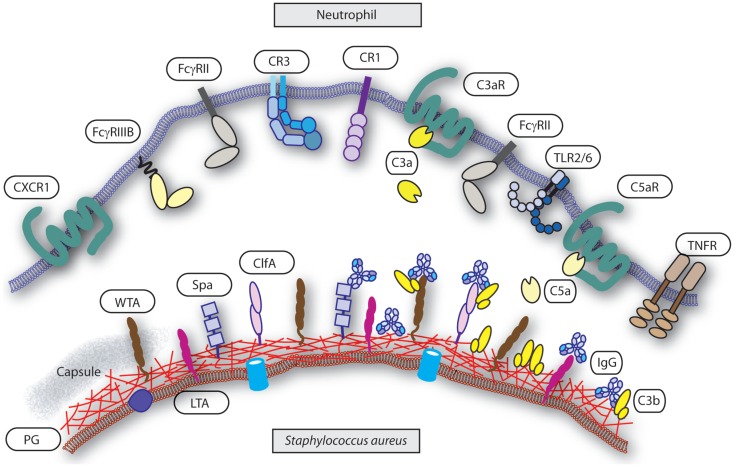
**Interface neutrophil and *S. aureus***. Several groups of receptors mediate neutrophils recognition of *S. aureus* upon opsonization and others are involved in activation or priming of phagocytosis. Targets on the *S. aureus* surface are the cell wall components peptidoglycan (PG), wall teichoic acid (WTA), lipoteichoic acid (LTA), capsule (“gray area”), and representative associated proteins clumping factor A (ClfA) and protein-A (Spa). Targets are decorated with serum derived opsonins IgG (binding with their Fab part) and C3b. Note the reverse Fc-dependent association of IgG with Spa. Receptors on the neutrophil surface involved in recognition of the opsonized *S. aureus* are FcγRII and FcγRIII for IgG, and CR1 and CR3 for C3b (and iC3b). Examples of receptors on the neutrophil involved in priming or activation of phagocytosis are complement receptors C3aR for C3a and C5aR for C5a, CXCR1 for il-8, and TNFR for TNFα. The heterodimer TLR2/TLR6 represents a common pattern recognition receptor for bacterial lipoproteins.

For neutrophils, initiation of phagocytosis requires decoration of bacteria with opsonins that are recognized by specific surface receptors. Igs and complement components are the predominant factor in serum that enables efficient opsonization. They are deposited on the surface of the bacterium and enable recognition by Fc receptors (FcRs) and complement receptors (CRs), respectively, which trigger the phagocytic machinery (Figure 1). Therefore, the interface between the specific opsonins and their receptors dictates phagocytosis and is influenced by both the target (bacterium) and effector (neutrophil) characteristics. Indirectly, the availability and nature of the opsonins are major determinants in the process but are dependent on the target bacterium, and the generation of specific Igs is part of the adaptive immune response and depends on antigen presentation, memory, and affinity maturation.

The most logical target candidates are of course surface-exposed proteins and general cell wall components, like peptidoglycan and wall teichoic acid (WTA) (15, 16). Since most humans are exposed to staphylococci already early in life without causing serious diseases, common structures, and/or proteins present on most staphylococci generate adequate Ig levels in normal healthy people. These structures provide sufficient natural occurring IgG that mediate recognition through FcγRs and also initiate classical pathway complement activation and thereby increase the amount of surface-bound opsonins, enabling uptake by neutrophils. Although the individual IgG levels by themselves do not seem to be that high, the power of the host defense is the combination of multiple IgG against several target molecules with complement activation. Lectin and alternative pathway-mediated complement activation results in the deposition of C3b/C3bi on the bacterial surface that is recognized by neutrophil CRs. Although phagocytosis is initiated, this system seems not to be so efficient in promotion of uptake of bacteria. On the other side of the spectrum, antibodies alone, and more specifically IgG, do trigger the FcγRs on the neutrophil to ingest the bacteria. For opsonization, location of the bound antibodies in relation to complement deposition and presentation to the neutrophil FcRs and CRs influences the opsonophagocytic potential. Thus, the efficacy of phagocytosis depends on the presence of specific IgG that activates the complement system via classical pathway resulting in C3b/C3bi deposition and binding to the FcγRs. The combination of these two key opsonins, complement, and IgG, triggers the phagocytic machinery into a high speed mode (17).

The attributed role of opsonins in neutrophil-mediated phagocytosis may, however, be dependent on the methodology, related to the site of infection. A recent example (18) showed that phagocytosis of *S. aureus* by human neutrophils in suspension depends on opsonization, while adherent neutrophils internalize more bacteria independent of opsonization. Consequently, addition of specific IgG did not enhance uptake by adherent neutrophils but improved the process in suspension. Remarkably, killing of the bacteria was only evident with adherent neutrophils when serum-induced aggregation phenomena were excluded. Under suspension conditions, persistence of free extracellular bacteria contributed substantially to the poor killing.

## Immunoglobulins and Their Receptors in Recognition of *S. aureus*

Immunoglobulin is the second most abundant protein in serum/plasma after albumin and provides life-long protection against infectious agents. Igs are important for proper opsonization of bacteria and subsequent recognition by specific FcR on the surface of phagocytes. Several Ig subtypes exist and they display specific functions; while IgG (IgG1, IgG2, IgG3, and IgG4), IgM and secretory IgA of the mucosal tissues have roles in infection control, IgE does not. Different IgG subclasses bind different FcγRs. Normal peripheral blood neutrophils express FcγRII (CD32) and FcγRIIIB (CD16), and do not express the FcγR1 (CD64) that is found on monocytes. However, during systemic infections and sepsis neutrophils do express FcγRI. Together with other activation-related surface antigens, expression of CD64 is used as specific sign of bacterial infections and marks the activation status of the neutrophil (19, 20). Most phagocytes including neutrophils express specific receptor for IgA (FcαR or CD89). When appropriate specific IgA antibodies are present, the FcαR initiated responses are comparable with those of IgG and FcγRII. In contrast to IgG, IgA does not activate the complement system. The binding characteristics between different Ig classes and the various FcRs are well documented; please refer to some excellent reviews (21–23).

For proper Ig opsonization, generation of target-specific Igs is crucial. In the case of *S. aureus*, effective Igs are directed against its surface molecules. The outer surface of Gram-positive bacteria, including *S. aureus*, contains a thick layer of peptidoglycan that is decorated with covalently anchored proteins that can initiate both IgG and complement deposition. The cell wall-associated proteins are often virulence factors and have been shown to influence pathogenesis (24). Naturally occurring antibodies directed against these proteins, like clumping factor A (ClfA) and protein-A, circulate in human serum, and several of these proteins are used as vaccine candidates to elicit protective antibodies. The presence of specific naturally occurring IgG directed against several surface-exposed cell wall components and proteins of *S. aureus* has been described by several studies that demonstrate that IgG provides the host with a handle to recognize and phagocytose the bacterium.

Much research has been performed on the efficacy of antibodies directed against specific staphylococcal targets. However, several studies also document a larger approach. Using a bead-based Luminex screening against 56 staphylococcal antigens – including many known virulence factors, surface-exposed, and secreted proteins, but also peptidoglycan and WTA – IgG and IgA levels in multiple serum samples of patients with a *S. aureus* bacteremia and non-infected controls were compared (25). IgG directed against all antigens were detected in healthy controls as well as patients at the time of diagnosis. In the majority of bacteremia patients, the IgG levels showed a temporal increase. Using classical ELISAs on 19 staphylococcal cell surface and secreted proteins, a wide range of antibody levels in both healthy donors and patients was observed (26). Here, the majority of IgG was induced by lipoteichoic acid (LTA) and peptidoglycan. The amount of anti-staphylococcal antibodies accounted for 0.1–3% of total serum antibodies and was already obvious in healthy adolescents aged 13–15 years. The total anti-staphylococcal IgG levels correlated with a functionality opsonophagocytosis assay using fluorescently labeled bacteria and P388.D1 mouse macrophage cells. A comparable ELISA screening study (27) was performed against 11 different purified antigens from *S. aureus*, including surface antigens (such as teichoic acid and clumping factors A and B) and secreted proteins (such as alpha-toxin, lipase, enterotoxin A, toxic shock syndrome toxin, scalded-skin syndrome toxin, fibrinogen-binding protein, and extracellular adherence protein). Most healthy individuals (15–89 years) had circulating IgG antibodies against these antigens, whereby titers against teichoic acid were the highest. An alternative strategy to demonstrate the antibody repertoire used 2-D immunoblots with sera from patients with *S. aureus* bacteremia on the extracellular protein profile of the infecting *S. aureus* strain (28). Pre-existing antibodies were present and the pattern of antibody response was distinct for carriers and non-carriers, but also did contain a common signature.

In addition to the wide studies pertaining to groups of *S. aureus* surface epitopes, much research has been done on specific staphylococcal antigens, i.e., peptidogylcan, the cell WTA (a glycopolymer covalently linked to peptidoglycan), polymeric-*N*-acetylglucosamine (PNAG) (29, 30), *N*-acetylglucosamine (GlcNac) (31), iron-regulated surface protein B (IsdB) (32, 33), and ClfA (34, 35). As an example, interesting studies have been published on the opsonizing capacity of peptidoglycan. Elevated IgG levels to peptidoglycan were observed in patients with deep tissue infection with *S. aureus* but hardly in patients with superficial staphylococcal infection (36). Peptidoglycan appeared to be the key cell wall component involved in staphylococcal opsonization, both by IgG and activating complement (37). By itself, peptidoglycan and its soluble parts are also potent activators of the host defense leading to pro-inflammatory cytokine release from monocytes (38) and oxidative burst in neutrophils (39), only valid for the polymeric form of peptidoglycan. In addition, small dipeptides or tripeptides derived from polymeric peptidoglycan trigger cytoplasmic nucleotide oligomerization domain (NOD) receptors leading to NF-kB activation (40). For *Bacillus anthracis*-derived peptidoglycan it has been shown that specific IgG mediates bacterial binding to monocytes and neutrophils. All tested healthy donors contained IgG recognizing peptidoglycan. The authors suggest that IgG facilitates uptake of complex peptidoglycan, where after small derivatives trigger NODs for cytokine release (41). Alternatively, the pentraxin serum amyloid P binds to peptidoglycan on the surface of *S. aureus* (in the absence of WTA in a dTagO strain) and subsequently to FcγR, which induces complement and IgG-independent phagocytosis (42). Notably, another pentraxin, C-reactive protein, was ineffective under identical conditions, and the presence of WTA seems to prevent this process. Pentraxins are known to bind and activate FcγRs in a structural similar fashion as IgGs do (43). In addition to Igs and pentraxins, mannose binding lectin (MBL) also recognizes peptidoglycan resulting in cytokine release from macrophages (44). WTA is also recognized by MBL and functions as an opsonins but only when anti-WTA IgG are not yet present like in infants (45). On the other hand, affinity purified anti-WGA IgG induces classical complement-mediated opsonophagocytosis of *S. aureus* (46). Using glycosyltransferase KO/mutant strains, beta-GlcNAc residues of WTA are shown to be required for the induction of the classical complement pathway-dependent opsonophagocytosis of *S. aureus*. MBL recognized both α- and β-GlcNAc residues of WTA. In normal human serum, the major antibodies to WTA are specific to β-GlcNAc-modified form (47).

Next to IgG, natural occurring IgM antibodies activate complement and thereby also have opsonophagocytic capacity. IgM against *S. aureus* is present in human serum as was demonstrated by a study using a multiplex Luminex assay 34. The level of *S. aureus*-specific IgM was relatively low, and IgM was directed mostly to microbial surface components recognizing adhesive matrix molecules (MSCRAMMs). When healthy donors and patients with infections were examined by ELISA, IgM antibodies to peptidoglycan were not detected (36). Dimeric IgA linked via the J chain and monomeric IgA found in serum are good opsonins for bacteria and promote neutrophil oxidative burst and phagocytosis as efficient as IgG (48).

Because IgG is of the highest importance for *S. aureus* opsonization, many studies focus on the identification of likely antigens for possible therapy. Some studies have used intravenous immunoglobulin (IVIgG) as a source to identify new IgG targets. IVIgG is a pool of IgG isolated from hundreds of healthy blood donors that is commonly used as replacement therapy in immunocompromised patients. The broad antibody spectrum present in such a pool provides protection against otherwise life-threatening infections and autoimmune diseases (49). Subtractive proteome analysis of *S. aureus* anchorless cell wall proteins was performed by screening for proteins reacting with IVIgG but not with IVIgG depleted of *S. aureus*-specific opsonizing antibodies. Three of the 40 candidate proteins, i.e., enolase, oxoacyl reductase, and hypothetical protein hp2160, were used as vaccine candidates in mice and provided protection. Affinity isolation of the IgGs from IVIgG resulted in opsonization, phagocytosis, and killing of *S. aureus* by human neutrophils (50). In addition, on studies using IVIgG, others have applied bioinformatics for antigen prediction selecting for lipoproteins and cell wall-anchored proteins from *Streptococcus sanguinis*. Eight protein candidates were randomly picked, pooled, and used to immunize rabbits. From the raised antibody pool, affinity chromatography demonstrated opsonic activity for the individual IgGs with human neutrophils and rabbit complement (51). Using DNA bar-coding and deep sequencing of IgG genes from *S. aureus* infected humans identified protective antibodies that promoted opsonophagocytosis (52).

## Complement Activation and Recognition during *S. aureus* Infection

The complement system is part of the innate immune defense and is a complex regulated system of proteases leading to soluble and surface-bound factors that contribute to efficient phagocytosis (53). Complement activation proceeds via three pathways, all leading to the cleavage of C3 into surface-deposited C3b (that also gets fragmented to iC3b) and released soluble mediator C3a. The “classical” activation of complement is mediated by specific antibodies bound to the bacterial surface that activate C1. Bacterially exposed carbohydrates are recognized by host lectins, mainly MBL, initiating the “lectin pathway.” Spontaneous and/or surface antigen-specific activation of C3 is possible as well as an amplification of the already-deposited C3b through the “alternative pathway.” The surface-bound C3b convertases activate new C3 molecules leading to more deposition of C3b onto the bacterial surface. The binding of C5 to the surface-bound C3b convertases subsequently initiates the release of the soluble mediator C5a and the formation of the membrane attack complex composed of C5b, C6, C7, C8, and multiple C9 molecules. The pores formed by the membrane attack complex may directly induce lysis of susceptible of mainly Gram-negative bacteria. The soluble cleavage products C3a and C5a, also known as anaphylatoxins (54), are chemoattractants and activators for phagocytes that express the C3aR and C5aR. With respect to phagocytosis, the bacterial surface-bound C3b is a key opsonin and is recognized by CRs on neutrophils. Both the complement factors as their receptors play several roles in phagocytosis. As the classical pathway is dependent on IgG, which was discussed in the previous section, more emphasis will be placed on other complement opsonin strategies in this section. Important group of CRs on neutrophils are lectins, those for bacterial surface-bound C3b or iC3b that belong to the adhesion receptor family and those for the soluble anaphylatoxins C3a and C5a that are G protein-coupled receptors (GPCRs).

Receptors that recognize surface-deposited complement factors are CR1 (CD35), CR2 (CD21), CR3 (CD11b/CD18 or MAC-1), CR4 (CD11c/CD18, integrin αXβ2), and CR of the Ig superfamily, CRIg (55). They all recognize C3b and/or iC3b, through distinct binding sites. Neutrophils express CR1 and CR3, whereas, macrophages express CR3 and CR4. CRIg is found on monocyte-derived macrophages and liver Kupfer cells (56). CR1 is a membrane glycoprotein with specificity for the complement products C3b, C4b and, with lower affinity, iC3b. It shares structural similarities with several regulators of complement activation, and the extracellular domain of CR1 consists of an array of 30 or more homologous units (57, 58). CR3 and CR4 are heterodimeric glycoproteins with a shared β-chain (CD18). Both receptors show specificity for the iC3b fragment (59, 60). Finally, CRIg is shown to mediate efficient phagocytosis of complement-opsonized particles and participates in the initial stage of phagosome formation (56).

Three cell-associated proteins have been described that show affinity for complement factor C1q, which is deposited upon recognition of surface-bound IgM. cC1qR, which closely resembles calreticulin, binds the collagen-like tail of C1q. C1qRp is a phagocytosis-promoting receptor that has similar ligand specificity, and gC1qR recognizes the globular head regions of C1q. Receptors for C1q are thought to play a role in both triggering and regulation of complement activation, and neutrophils express all three types of receptors (55, 61).

Pertaining to complement and specific recognition of *S. aureus*, although all three complement pathways play a role, lectins have thoroughly been investigated. Direct binding of MBL to several micro-organisms, including *S. aureus*, was demonstrated by flow cytometry, and bound MBL promoted deposition of C4 (62). The contribution of the MBL–MASP pathway relative to other pathways of complement activation on *S. aureus* and the consequence for C3b deposition and phagocytosis was demonstrated using MBL-deficient sera (63). MBL–MASP added to deficient sera enhanced the generation of C3b and iC3b on the surface of staphylococci and subsequently increased phagocytosis by human neutrophils. It must be noted that others have observed that while yeast species are preferentially opsonized and subsequently phagocytosed via activation of the lectin pathway of complement, the uptake of bacterial strains, including *S. aureus*, was largely MBL independent (64). Using MBL-deficient sera, it was shown that there was less C3 deposition on zymosan and Candida, while C3 deposition was not different on *S. aureus* using MBL-sufficient and MBL-deficient serum. Same authors have shown that inhibition of the classical pathway of complement activation during opsonization with either MBL-sufficient or MBL-deficient sera induced a two to threefold reduction in the subsequent phagocytosis of *S. aureus*. An alternative role for MBL in *S. aureus* infection is suggested independent of opsonization or complement activation. Binding of MBL to LTA via its carbohydrate recognition domain and subsequent complexing with TLR2 to increase ligand delivery is described to enhance TLR2 responses, as was measured by cytokine release by murine macrophages. This TLR2-mediated responses were only effective when *S. aureus* was delivered into the phagosome (65).

In addition to MBL, other sugar pattern recognition molecules as ficolins and collectins play a part in the lectin complement pathway and aid in *S. aureus* recognition. They both contain lectin activities within the C-terminus. Ficolins consists of collagen-like long thin stretches and fibrinogen-like globular domains with lectin activity, usually specific for *N*-acetylglucosamine (GlcNAc) (66). L-ficolin specifically binds to LTA, and immobilized LTA from *S. aureus* binds L-ficolin complexes from sera. These complexes are described to initiate lectin pathway-dependent C4 turnover (67). Collectins are a family of proteins that contain both collagen-like regions and lectin domains and play a role in opsonization and activation of neutrophils. In addition to MBL, conglutinin and the surfactant proteins SP-A and SP-D, which participate in the innate immunity of the lung, are other members of this group. The collectins share structural similarities with complement component C1q. SP-A may act directly as an opsonin for micro-organisms by binding via their lectin domains and enhance phagocytosis by alveolar macrophages. In addition, SP-A directly binds to C1q and thereby facilitates uptake of C1q-coated targets (68). SP-A and SP-D enhance bacterial (*Escherichia coli*, *S. pneumoniae*, and *S. aureus*) uptake by human neutrophils through a mechanism that involves both bacterial aggregation and direct actions on neutrophils. Multimerization of SP-D is an important parameter in its ability to increase uptake and differs thereby from the classical opsonins IgG and complement (69). Sp-A enhances the uptake of IgG-coated polystyrene beads by inflammatory neutrophils via direct interaction with both the opsonized beads and the neutrophils. In this case, the rat neutrophils were obtained from a lavage of LPS challenged lungs, and here SP-A did not have a generalized activation effect on neutrophils (70).

Finally, an important group of CRs on neutrophils are those for the soluble anaphylatoxins C3a and C5a. The C3a receptor (C3aR) and C5aR belong to the family of GPCRs. Neutrophils express a large number of GPCRs that participate in the host defense. They can sense bacterial peptides and toxins, e.g., formyl-peptide receptors (FPR1 and FPR2), lipid mediators, e.g., leukotriene B4 receptor and platelet activating factor receptor, and chemokine receptors (71, 72). Although known for their role in migration of neutrophils, most of these ligands, including C3a and C5a, also trigger direct cell activation, like ROS production, or “prime” cells for subsequent activation by other agonists. A crucial role for C5a–C5aR interaction was demonstrated for *E. coli*-induced phagocytosis in lepirudin anticoagulated whole blood. Generation of the anaphylatoxin C5a was essential and preceded the up-regulation of CR3, which was required for the subsequent oxidative burst and phagocytosis (73). Also for *S. aureus* in lepirudin treated whole blood, complement was responsible for phagocytosis as well as leukocyte activation (74).

## Neutrophil Priming

Neutrophil priming is a process that causes a dramatic increase in the response of the cells and allows for faster and more efficient response, including phagocytosis of invading pathogens (12, 75). Activating agents have direct effects on cell surface receptor expression, but also intracellular in superoxide anion generation, degranulation, and mediator release (70). In the primed state, there is no increase in oxidase activity, yet subsequent stimulation provokes a response that is larger than in non-primed, activated cells. Neutrophil priming for enhanced oxidative burst, phagocytosis, and killing is initiated by several agents, such as soluble complement activators C3a and C5a (74), interferon-γ (IFNγ) (76, 77), interleukin-8 (il-8) (78), tumor necrosis factor-α (TNF-α) (79), and granulocyte/macrophage colony-stimulating factor (G-CSF & GM-CSF) (80). Stimulating receptors for G-CSF, GM-CSF, and IFNγ also delay apoptosis thereby affecting survival of neutrophils (81).

As mentioned, sensing of C5a is important in priming neutrophils by inducing up-regulation of CR3, which is crucial for subsequent phagocytosis in whole blood. Regarding TNF-α, neutrophils express several members of the TNF receptor family with diverse biological functions, including TNFR1 and TNFR2. TNF-α is an important pro-inflammatory cytokine that both directly activates neutrophils and primes the cells for subsequent stimulation including phagocytosis (82). Interleukin-1 plays also a major role in the inflammatory response and *S. aureus* infections and triggers a modest neutrophil activation (83, 84). They do express the IL-1R1, a member of the IL-1/TLR super family with comparable intracellular domains. The predominant receptor on neutrophils is the decoy receptor IL-1RII lacking intracellular signaling domains. Different classes of chemotactic agents, including C5a and il-8, cause a rapid reduction in the IL-1 binding capacity by human neutrophils (85).

Neutrophils express several innate immune receptors that are involved in recognition of pathogens and danger signals and allow priming of the cells. Those are found on the cell surface as well as in intracellular endocytic compartments and lead to cell activation, priming, and transcriptional changes. Neutrophils express most of the known TLRs that recognize bacterial structures, most importantly TLR4, which recognizes lipopolysaccharide from Gram-negative bacteria, and TLR2, which recognizes lipoproteins like LTA from Gram-positive bacteria. Stimulation of neutrophils with several TLR ligands stimulates phagocytosis of latex beads (86) and enhance the oxidative burst (87). Neutrophils also express members of the C-type lectins, such as Dectin-1, the receptor for fungal β-glucans and the components of the NLRP3 inflammasome that include the cytoplasmic NOD-like receptors sensing bacterial proteoglycan degradation products and danger signals (88). TLRs are not phagocytic receptors *per se*, but they are also internalized in the process and therefore participate to the link between phagocytosis and inflammatory responses by triggering the production of cytokines.

## Phagocytosis

Phagocytosis is defined as the engulfing of other cells, cell fragments, and micro-organisms. Thereby it leads to sequestration and elimination of e.g., dead cells after apoptosis, pathogens like bacteria and uptake or delivery of substances by therapeutic micro- or nanoparticles. Physical parameters, i.e., particle size, shape, deformability, as well as biological parameters determine the effectiveness of phagocytosis.

**Figure 2:**
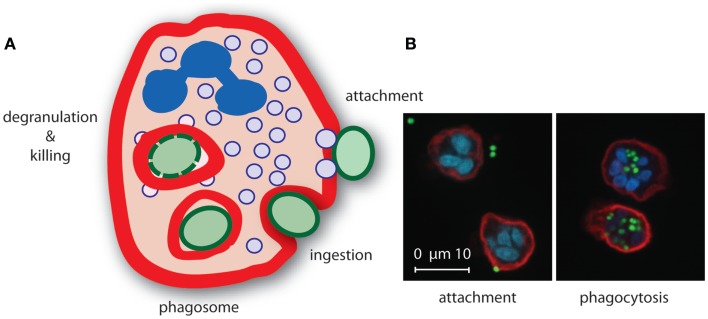
**Neutrophil phagocytosis of *S. aureus***. **(A)** Upon recognition of opsonized *S. aureus*, neutrophils internalize the bacterium in a phagosome where secretory granule content is released and ROS are produced that mediate killing of the bacterium. **(B)** Confocal microscopy image of *S. aureus* expressing green fluorescence protein (green) attached (left) to and phagocytosed (right) by neutrophils [red, membrane stain wheat germ agglutinin (WGA)-Alexa647, blue, nucleic acid stain Syto82].

The actual phagocytosis process can be distinguished in several phases: (1) attachment of the opsonized particle upon recognition by specific receptors, (2) pseudopod extensions around attached particle whereby it is still exposed to the environment (“zippering”), and (3) completion of the engulfment resulting in the formation of a phagosome, which is an outside-in compartment inside the cell (Figure 2). The next steps involve mobilization and fusion of the phagosome with different granule types resulting in the liberation of granule content that is required for killing of the microorganism (89, 90). Concurrently, a strong oxidative burst is initiated in the phagosome by NADPH-dependent oxidases upon triggering of specific cell surface receptors, leading to the generation of highly toxic ROS. Together with the granular content, ROS play an important role in bacterial killing (91).

Neutrophils are capable of engulfing as many as 50 bacteria whereby a substantial amount of surface area is internalized while maintaining cell size and shape. For macrophages it is shown that the surface membrane internalized during engulfment is replaced from intracellular reservoirs. It is believed that during this replacement of the plasma membrane originating from endoplasmatic vesicles, focal exocytosis is effective leading to cytokine release. To form an autonomous internal compartment, the phagosome must undergo fission from the plasma membrane. The actual proteins involved in this process remain not fully understood, but involve dynamin and myosins. An important notion for any phagocytosis process is that not all phagosomes are created equal (89).

Besides the appropriate opsonins and their ligands, neutrophil versus monocytes/macrophages are of influence on this process, also physical parameters have their influence, like target particle size (>5 μm) and even their shape as shown for *S. pneumoniae*. Increasing the bacterial chain length, by natural changes in cellular morphology or via antibody-mediated agglutination, promoted complement-dependent killing (92). The density of both the ligand, e.g., IgG bound to the surface of the target particle, and the receptor, e.g., FcγR, also influence phagocytosis. Using RAW 264.7 murine macrophage cell line, IgG density had different effects on uptake depending on the particle size (93). Phagocytosis and IgG-mediated triggering are sometimes misleading if immune complexes are used to stimulate cells. Although these complexes could be considered as very small particles, the nature of these complexes differs from that of e.g., bacteria. Others call opsonized particles immune complexes. By loading sheep red blood cells (ShRBC) with increasing amounts of IgG, the increase in IgG density corresponds with increase in phagocytosis by mouse macrophages, both in number of positive cells and ShRBC per cell (94). For high-throughput assays, automatic imaging methods or flow cytometry are becoming more popular and rely on fluorescent beads and cell lines in microplate formats (95, 96).

Neutrophils are extremely efficient phagocytes and can internalize IgG-opsonized latex beads in <20 s (97). Localized granule secretion is important for phagocytosis and the generation of an anti-microbial phagosome. Phagocytosis of IgG-opsonized zymosan particles was very fast and most phagosomes were sealed within 1–3 min (98). Fusion of azurophilic granules and specific granules with the plasma membrane preceded phagosome sealing, and the granule membrane markers (CD63 and CD66b) were observed at the site of phagocytosis. Whereas, azurophilic granules are mainly secreted toward the forming/formed phagosome, specific granules can fuse with the plasma membrane at any location. A consumption of protons increases the pH, and early studies even indicated alkaline levels in early phagosomes before the pH gradually decreased (90, 99). It must be noted that during phagocytosis several signaling pathways are initiated that direct cellular activities but are beyond the scope of this review (81, 100–103).

## Killing Process

The major task for neutrophils in the host defense is the elimination of pathogens by efficient uptake and subsequent killing. Therefore, neutrophils are equipped with an arsenal of anti-bacterial products stored in granules and an efficient massive generation of ROS by the membrane-associated NADPH-oxidase that can kill microorganism upon phagocytosis. In addition, neutrophils can kill bacteria extracellularly by release of chromatin covered with granule contents and selected cytoplasmic constituents providing NETs.

Neutrophils contain several granule subtypes that are subdivided into peroxidase-positive granules [containing myeloperoxidase (MPO)], which are also called primary or azurophil granules, and peroxidase-negative granules termed specific or secondary granules. However, granules are much more heterogeneous with subsets defined by a selection of marker proteins (104). MPO is one of the most abundant proteins in the neutrophil azurophil granules and catalyzes the oxidation of halide ions in the presence of hydrogen peroxide and generates hypochlorous acid that aids in bacterial killing (105). The granules contain a broad-spectrum of bactericidal and degradative proteins, but also growth depriving factors. Lactoferrin binds iron and neutrophil gelatinase-associated lipocalin (NGAL) interferes with siderophore-mediated iron acquisition thereby sequestering an essential microbial growth factor (106). Lysozyme hydrolyzes β (1–4) glycosidic linkages in the peptidoglycan layer, compromising bacterial integrity. Cationic anti-microbial peptides (CAPs) such as defensins and cathelicidin-type peptides (LL37) bind with high affinity into the bacterial membrane causing disruption (107–109). Azurophil granules also contain a family of structurally related serine proteases, cathepsin G, elastase, and proteinase 3, with anti-microbial and regulatory activity (110, 111).

The neutrophil NADPH-oxidase is a multicomponent enzyme complex that transfers electrons from NADPH onto molecular oxygen, thereby generating superoxide anion. Spontaneous dismutation together with the granule-derived MPO and iron-catalyzed reactions leads to the formation of several reactive ROS that include hydrogen peroxide, hydroxyl radicals, hypochlorous acid, and singlet oxygen. The transport of electrons is mediated by the redox center that contains Cytochrome b558, a flavo-hemeprotein composed of two subunits, gp91^phox^ and p22^phox^. Assembly of the NADPH-oxidase involves translocation of three soluble components, p47^phox^, p67^phox^, p40^phox^, and a GTP-binding protein onto the plasma membrane to form the complete oxidase. This complex system is also carefully regulated in place and time, and it is concurrently assembled with phagosome formation (91). Specific KO mice are used to study the contribution of the NADPH-oxidase complex in bacterial pathogenesis and survival of the host. The oxidase response of these KO neutrophils is severely reduced upon stimulation with particles (IgG latex beads or *S. aureus*), soluble mediators like fMLF, or is adhesion dependent. Phagocytosis of bacteria by KO neutrophils is normal, but a clear defect is observed in killing of *S. aureus in vitro* as well as *in vivo* (112). Both reactive oxygen metabolites and neutrophil serine proteases contribute to the host defense depending on the pathogen studied, as was shown in the case of *Aspergillus* (111). The importance of an intact oxidative burst in neutrophils is demonstrated by chronic granulomatous disease (CGD; a primary immunodeficiency disorder of phagocytes) patients with a genetic defect in one of the NADPH-oxidase subunits. These patients suffer from recurrent bacterial infections, most commonly *S. aureus*, *Aspergillus* spp, and *Salmonella* spp (113). The role of ROS in killing of bacteria by neutrophils *in vitro* is evident when cells are treated with diphenyleneiodonium (DPI), which binds strongly to flavoproteins and is thereby a powerful inhibitor of several important enzymes including the NADPH-oxidase. Another defect is found for MPO deficiency; patients are fairly asymptomatic and defects are mainly found in the formation of NETs (9, 114). The oxidative burst pumps electrons into the phagosome that is compensated by a flux of K^+^ ions across the membrane in a pH dependent matter. This is an important trigger for the release of cationic granule proteins (115, 116).

Oxidative deamination of L-arginine by nitric oxide (NO) synthetase generates NO that together with superoxide anion forms reactive nitrogen intermediates with anti-microbial activity. The production of NO within phagocytes is an important component of the innate immune response to infection, and requires inducible NOS (iNOS). In mice macrophages are the source for NO, but in human leukocytes levels of iNOS are far more regulated (105, 117, 118). The role of iNOS in human neutrophils is limited and requires cytokine activation (119).

For effective bacterial killing, a critical concentration of neutrophils is required in suspension. Mathematical models have been applied to examine this ratio, which was supported by experimental data. By varying both neutrophil and bacterial concentrations (*S. epidermidis*), it was documented that the killing rate requires a critical neutrophil concentration of 3–4 × 10^5^ per ml, is independent of ratio neutrophils to bacteria, and fitted an exponential function. The mathematical model fits the killing of Gram-positive and Gram-negative bacteria, opsonized by IgG and C3, by neutrophils in suspension (120). Further mathematical modeling and *in vitro* experimental killing of serum-opsonized *S. aureus* demonstrated that a critical neutrophil concentration dictates the outcome. Here, the individual maximum bearable bacterial concentration depended on neutrophil concentration, phagocytic activity, and patient barrier integrity and varied by orders of magnitude between patients (121).

Neutrophils can kill bacteria extracellular by release of NETs that trap bacteria covered with anti-microbials. This release and formation of NETs is the last step in an active neutrophil death termed NETosis and requires the formation of ROS. Several different agonists trigger the NET formation, including cytokines, microbial components, and bacteria itself. The extracellular traps are described to be formed *in vivo* and to contribute to clearing of infections (13, 122). In response to *S. aureus*, neutrophils employ a very rapid (5–60 min) alternative process of NET formation, independent of ROS, employing budding of vescicles. This response requires live bacteria and was mimicked by Panton–Valentine leukocidin as soluble mediator (123). Another member of this pore-forming leukotoxins, LukGH (also known as LukAB), also promotes the release of NETs (124). IgA-opsonized *S. aureus* also rapidly initiates the formation of NETs probably due to the robust production of ROS. In these circumstances killed bacteria or beads coated with IgA are also effective and engagement of the FcαR is a prerequisite (125).

Killing of *S. aureus* by neutrophils is not only effective in suspension but is also observed when bacteria are found in a biofilm. In contrast to mouse macrophages, neutrophils are capable of phagocytizing biofilm-associated *S. aureus in vitro* (126). Neutrophils migrate into the biofilm and enable clearance of bacteria by phagocytosis, whereby the extent of cleareance is dependent on the maturation state of the biofilm. Young developing biofilms are more sensitive toward the attack by neutrophils compared to more mature biofilms (127).

Opsonization with normal human serum did not change the phagocytosis of bacteria in biofilms, while opsonization of dispersed *S. aureus* was enhanced. IgG coating of the biofilm induced oxygen radical production that improved clearance of the biofilms due to bacterial killing (128).

## Anti-Opsonic/Phagocytic Strategies

Phagocytosis by neutrophils is a very effective mechanism for *S. aureus* clearance. However, staphylococci are not just mere bystanders, and the bacteria can employ several anti-opsonic and anti-phagocytic means to survive. Escape strategies can be found for every step of phagocytosis, from binding and cleavage of antibodies to escape from phagosomes.

The best way to prevent phagocytosis is to prevent the initial proper opsonization, either directly by taking a sugar coating (capsule) that covers most of the more antigenic/immunogenic surface-exposed proteins, or indirectly by employing decoy molecules and active removal of opsonins. A rather thick polysaccharide capsule is best known for the many pneumococcal serotypes, but also for staphylococci a capsular polysaccharide efficiently hinders phagocytosis by neutrophils. Up to 50% of clinical *S. aureus* isolates have a capsule or microcapsule divided in up to 11 serotypes, whereby most of them react with antibodies directed to capsule type 5 or 8. These bacteria resist opsonophagocytosis and hence killing when bacteria were grown under conditions for optimal capsule production. These capsule polysaccharides and their components are also used as candidates for vaccine development against staphylococci. Specific antibodies directed against the capsule provide efficient opsonization and thereby uptake by neutrophils (129, 130). Recently an indirect covering of *S. aureus* with a shield of fibrinogen is shown by the staphylococcal extracellular fibrinogen-binding protein (Efb) that links the surface C3b deposition with fibrinogen. Thereby Efb prevents binding of phagocytic receptors to the opsonins C3b and IgG and subsequent phagocytosis (131).

Another anti-opsonic strategy is the secretion of IgG-binding proteins. The best known IgG-binding protein is staphylococcal protein-A (SpA). This ubiquitously expressed protein is a prominent cell wall-anchored protein, but it is also found in the supernate. Typically, its C-terminal part is linked to the cell wall, while the N-terminus contains five highly homologous extracellular Ig-binding domains in tandem. Each domain binds Igs through the Fc and restricted Fab domain of the human heavy chain VH3 family. The residues involved in binding to Fab are distinct from the residues that mediate binding to Fc part as the Fab fragments through distinct residues (132). Fc-binding to SpA is thought to protect staphylococci from opsonophagocytic killing (133–135). However, the precise mechanism is still not clear. Binding of Ig is essential though for *S. aureus* escape from host immune surveillance in mice as a protein-A-deficient strain induces a less severe arthritis and septic death, indicating that protein-A is a virulence factor (136). *S. aureus* also contains another IgG-binding protein, *S. aureus* binder of IgG (Sbi) that exhibits two Ig-binding domains similar to those of protein-A with comparable specificity (137, 138). Sbi is a multifunctional bacterial protein, which also acts a complement inhibitor and interferes with innate immune recognition (139). In a whole blood assay, Sbi prevents neutrophil-mediated opsonophagocytosis thereby promoting bacterial survival. The Sbi-KO bacteria show increased survival in whole blood and better uptake by neutrophils (140). However, Sbi does not contribute to *S. aureus* virulence in contrast to the Fcγ and VH3-type Fab binding activities of SpA (141). The impact of “reverse” IgG-binding on the interaction with host defense and invasive infection is shown for streptococcal protein M1 and H. Antibodies bound via Fab facilitate opsonization and killing by neutrophils whereas Fc-binding to protein M and H protected against phagocytosis (142).

Another strategy to escape killing, is through cleavage of IgG and C3, stretegies predominantly described for streptococci. Staphylokinase (SAK) is a secreted protein that can form a complex with human plasminogen resulting in the formation of active plasmin, a broad-spectrum proteolytic enzyme. This facilitates bacterial penetration into the surrounding tissues and is shown to cleave bacterial surface-bound IgG and C3b molecules. Cleavage of these important opsonins results in diminished uptake of the bacteria by neutrophils (143). Both staphylococcal proteins Sbi and Efb bind simultaneously C3/C3b and plasminogen on the surface of the bacterium and thereby enable the recruited plasmin or SAK to cleave bound C3 and C3b as well as soluble C3a (144).

In addition to strategies targeting IgG for anti-phagocytic purposes, staphylococci secrete many complement inhibitors to halt effective phagocytosis. These include, amongst others, staphylococcal complement inhibitor SCIN, extracellular complement-binding protein Ecb, and staphylococcal superantigen-like protein SSL7. They generally affect conversion of complement through binding major complement convertase componants and halt the complement cascade at several stages. As staphylococci have a large arsenal of complement inhibitors, please refer to excellent overviews (3, 145–147).

In addition to anti-opsonic strategies, bacteria, including *S. aureu*s, have developed strategies to evade killing after internalization. Staphylococci can survive within the phagosome and have developed several ways to escape neutrophil killing. Escape is accomplished by employing toxins, like phenol-soluble modulins and leukocidin AB, that lyse the neutrophil from within after phagocytosis (148, 149). Toxin production in this bacterium is mainly regulated by activation of the *agr* operon (150), and the survival of the bacterium inside the neutrophil could even contribute to spread of the bacteria in the host (151). *S. aureus* also evades the ROS mediated killing mechanism using scavengers like catalase, superoxide dismutase (152, 153), and the golden carotenoid pigment (154). With regard to NETs, some bacteria can escape entrapment through secretion of endonucleases that can liberate them from the traps. An alternative NET escape mechanism for *S. aureus* is by converting NETs to deoxyadenosine, which induces macrophage cytotoxicity. Two secreted proteins, nuclease and adenosine synthethase are required for this host cell death (155).

## Concluding Remarks

For invading staphylococci, phagocytosis and killing by human neutrophils is the biggest threat. Neutrophils are the only cells that can effectively kill staphylococci by engulfment and subsequent bombardment with proteases, amidases, anti-microbial peptides, and proteins in concert with ROS that are generated during the metabolic burst. Both complement and antibodies are crucial for effective uptake and neutrophil activation. *S. aureus* is not an innocent bystander in this process. It actively secretes several proteins to impair every single step in this process from receptor modulation, to complement inhibition to neutrophil lysis to protease, anti-microbial peptide inhibition and resistance to ROS. For the design of future novel anti-microbial strategies: therapeutic antibodies, vaccines, and novel antibiotics, all this should be taken into account. Still the best way to treat diseases is to help to enhance the natural defense mechanisms that are already in place.

## Conflict of Interest Statement

The authors declare that the research was conducted in the absence of any commercial or financial relationships that could be construed as a potential conflict of interest.
